# Capillary refill time variation induced by passive leg raising predicts capillary refill time response to volume expansion

**DOI:** 10.1186/s13054-019-2560-0

**Published:** 2019-08-16

**Authors:** Matthias Jacquet-Lagrèze, Nourredine Bouhamri, Philippe Portran, Rémi Schweizer, Florent Baudin, Marc Lilot, William Fornier, Jean-Luc Fellahi

**Affiliations:** 1 Département d’Anesthésie Réanimation, Centre Hospitalier Louis Pradel, Hospices Civils de Lyon, 59 Boulevard Pinel, 69500 Bron, France; 2 Université Claude-Bernard, Lyon 1, Campus Lyon Santé Est, 8 avenue Rockefeller, 69008 Lyon, France; 3 Département de Réanimation Pédiatrique, Centre Hospitalier Femme mère enfant, Hospices Civils de Lyon, 59 Boulevard Pinel, 69500 Bron, France; 4 Département d’Anesthésie Pédiatrique, Centre Hospitalier Femme Mère Enfant, Hospices Civils de Lyon, 59 Boulevard Pinel, 69500 Bron, France; 5 Centre Lyonnais d’Enseignement par Simulation en Santé, SAMSEI, Université Claude Bernard Lyon 1, Lyon, France; 6 Health Services and Performance Research Lab (EA 7425 HESPER), Université Claude Bernard Lyon 1, Lyon, France; 7 EPICIME-CIC 1407 de Lyon, Inserm, Hospices Civils de Lyon, F-69677 Bron, France

**Keywords:** Capillary refill time, Fluid responsiveness, Passive leg raising, Peripheral perfusion, Microcirculation, Circulatory shock, PCO_2_gap

## Abstract

**Background:**

A peripheral perfusion-targeted resuscitation during early septic shock has shown encouraging results. Capillary refill time, which has a prognostic value, was used. Adding accuracy and predictability on capillary refill time (CRT) measurement, if feasible, would benefit to peripheral perfusion-targeted resuscitation. We assessed whether a reduction of capillary refill time during passive leg raising (ΔCRT-PLR) predicted volume-induced peripheral perfusion improvement defined as a significant decrease of capillary refill time following volume expansion.

**Methods:**

Thirty-four patients with acute circulatory failure were selected. Haemodynamic variables, metabolic variables (PCO_2_gap), and four capillary refill time measurements were recorded before and during a passive leg raising test and after a 500-mL volume expansion over 20 min. Receiver operating characteristic curves were built, and areas under the curves were calculated (ROC_AUC_). Confidence intervals (CI) were performed using a bootstrap analysis. We recorded mortality at day 90.

**Results:**

The least significant change in the capillary refill time was 25% [95% CI, 18–30]. We defined CRT responders as patients showing a reduction of at least 25% of capillary refill time after volume expansion. A decrease of 27% in ΔCRT-PLR predicted peripheral perfusion improvement with a sensitivity of 87% [95% CI, 73–100] and a specificity of 100% [95% CI, 74–100]. The ROC_AUC_ of ΔCRT-PLR was 0.94 [95% CI, 0.87–1.0]. The ROC_AUC_ of baseline capillary refill time was 0.73 [95% CI, 0.54–0.90] and of baseline PCO_2_gap was 0.79 [0.61–0.93]. Capillary refill time was significantly longer in non-survivors than in survivors at day 90.

**Conclusion:**

ΔCRT-PLR predicted peripheral perfusion response following volume expansion. This simple low-cost and non-invasive diagnostic method could be used in peripheral perfusion-targeted resuscitation protocols.

**Trial registration:**

CPP Lyon Sud-Est II ANSM: 2014-A01034-43

Clinicaltrial.gov, NCT02248025, registered 13th of September 2014

**Electronic supplementary material:**

The online version of this article (10.1186/s13054-019-2560-0) contains supplementary material, which is available to authorized users.

## Introduction

Shock is one of the most common life-threatening conditions in critical care and a frequent cause of admission to intensive care units [1, 2]. Variables related to macrocirculation, such as mean arterial pressure and central venous pressure, are used in the haemodynamic assessment of critically ill patients [1, 2]. These variables are considered as good surrogates to guide haemodynamic resuscitation [1]. However, macrohaemodynamic variables may poorly reflect tissue perfusion and microcirculation [3]. Lactate reflects tissue perfusion and lactate-targeted resuscitation is the gold standard under current guidelines [1, 2], but lactate increase can have various explanation, and its decrease can be prolonged compared to peripheral perfusion [4]. Peripheral perfusion evaluation reflects intra-abdominal visceral organ perfusion [5]. A mottled skin and an increased capillary refill time (CRT) attest peripheral perfusion. CRT is defined as the time taken for a distal capillary bed to regain its colour after pressure has been applied to cause blanching [6]. CRT has an acceptable prognostic value [7, 8]. Abnormal peripheral perfusion after initial resuscitation is associated with increased morbidity and mortality [9–11]. CRT is widely used in critically ill paediatric and adult patients [14, 15]. Some authors praise to use peripheral perfusion-targeted therapy [12, 13]. Peripheral perfusion-targeted resuscitation is enticing as it might provide a real-time response to increases in flow. This could accelerate the decision to stop resuscitation and avoid the risks of fluid overload [14]. Recent studies have tested this hypothesis but have failed to show superiority against lactate-targeted resuscitation [15]. Many studies have focused on the prediction of cardiac index (CI) changes of a volume expansion while few have investigated the effects of volume expansion on tissue perfusion [16]. Passive leg raising (PLR) predicts fluid responsiveness based on cardiac output changes [17]. PLR has also been reported as a surrogate of volume expansion to assess the effect of volume expansion on the microcirculation [18]. As peripheral perfusion-targeted therapy is gaining importance, and other studies are expected in this scope [19], a direct prediction of the effect of fluid on peripheral perfusion improvement could be helpful to tailor further studies.

Therefore, we hypothesised that a rigorous protocol to measure CRT variation in association with standardised PLR would be discriminating to predict peripheral perfusion response to fluid in adult patients with circulatory shock.

## Materials and methods

### Ethics

The study protocol was approved by our Institutional Review Board (IRB) for human projects (CPP Lyon Sud-Est, ANSM: 2014-A01034-43), and the protocol was published a priori (Clinicaltrial.gov: NCT02248025). Oral and written information was given to all patients or relatives. Signed consent was waived by the ethics committee. To allow our readers to assess the risk of bias, we followed the Standards for Reporting Diagnostic Accuracy (STARD) statement to design and report the study [19].

### Patients

This prospective observational study was conducted in a 20-bed adult cardiothoracic intensive care unit in a tertiary teaching hospital (Louis Pradel Hospital) in Lyon between September 2014 and December 2016. All patients diagnosed with acute circulatory failure to whom the attending anaesthesiologist decided to administer a volume expansion could be included. Eligibility criteria were as follows: the patient required an arterial and a central venous catheter, a CRT had to be measurable, a cardiac output monitoring by transpulmonary thermodilution (PiCCO™ PULSION Medical Systems, Munich, Germany), and a 500-mL volume expansion needed to be prescribed by the attending physician. We defined acute circulatory failure according to the ESICM guidelines [1]. We excluded patients with the following characteristics: pregnancy, cardiogenic pulmonary oedema with acute respiratory failure, mechanical circulatory support, a moribund state, intra-abdominal hypertension, and lower limb amputation or compression stockings.

### Study protocol and measurements

The study protocol encompassed four steps: baseline (T1), during PLR (T2), at return to baseline (T3), and after volume expansion (T4). At each of these steps, the following macrohaemodynamic variables were collected: systolic, diastolic, mean, arterial, and central venous pressure; heart rate; and cardiac output (CO), and we calculated the cardiac index (CI) as CO divided by the body surface area. Four consecutive CRT measurements were assessed at each time T1, T2, and T4. Mottling score [11] and metabolic variables (arterial and venous blood gases including arterial lactate) were also collected at T1 and T4, enabling us to calculate oxygen delivery, oxygen uptake, venous-to-arterial difference in carbon dioxide partial pressure (PCO_2_gap), modified respiratory quotient, and oxygen extraction ratio (formulae of calculation detailed in Additional file 1: Annex 3). We collected respiratory rate and pulse oximetry, and in cases of mechanical ventilation, we also assessed end-expiratory pressure, plateau pressure, tidal volume, and pressure support variables (Sequential Organ Failure Assessment score [20] at inclusion and new Simplified Acute Physiology Score (SAPS2) [21]). Patients were a posteriori sorted into two groups: capillary refill time responders (CRT-R) and non-responders (CRT-NR), according to the reduction of at least 25% of CRT following volume expansion or not. Patients were also a posteriori sorted into two other groups: cardiac index responders (CI-R) and non-responders (CI-NR), according to the increase of at least 15% of CI following volume expansion or not.

We recorded CRT with a smartphone’s video camera iPhone 6™ (Apple Inc., Cupertino, CA) characteristics: 8-megapixel iSight™ camera with 1.5 micropixels, autofocus with focus pixels, ƒ/2.2 aperture with video recording (1080p HD video recording, time-lapse video with stabilisation, cinematic video stabilisation, 30 images/s, with locked continuous autofocus). We controlled lighting conditions using the flashlight system. We made a calibrated compression of the skin using a piston for seven seconds (Additional file 1: Annex 2). The piston characteristics were as follows: a 10-ml syringe (BD Plastipak™, Plymouth, MI) filled in with 10 ml of air and closed with a plug (Vygon™, Ecouen, France). We had chosen the duration of compression according to a previous publication [6], and the pressure was chosen to decrease intra-observer variability (personal data). We applied the piston on the skin; the 10 ml of air was compressed to fit a 7-ml volume, generating a pressure at the surface of the skin of 176 mmHg on a 2.5-cm^2^ surface (personal data). Four CRT acquisitions were made on the thorax at each haemodynamic condition in less than 3 min by a single investigator (MJL) and subsequently averaged and analysed a posteriori by 2 readers (MJL and NB) using the freeware Kinovea™ (www.kinovea.org). The video was seen several times to determine the end of the CRT, and the chronometer of the software was used to assess CRT. The readers were blinded to the clinical condition of the patients, to the evaluation of the index test (ΔCRT-PLR), and to the reference standard (ΔCRT-VE). In 20 patients, 4 CRT were analysed by 2 observers (MJL and NB) to evaluate both intra- and inter-observer reproducibility. As recommended, we performed PLR from a semi-recumbent position at 45° [22]. Volume expansion consisted of a 500-mL lactate Ringer administration over 20 min. No modifications to the administration rate or new drug administration have occurred in the study period.

### Endpoints

The primary endpoint of the study was to determine the diagnostic ability of ΔCRT-PLR to predict peripheral perfusion response, defined as a CRT decrease of at least 25% following volume expansion (VE). Secondary endpoints were to compare ΔCRT-VE, ΔCRT-PLR, metabolic and macrocirculatory variables, and prognostic markers and to measure the inter- and intra-observer variabilities.

### Statistical analysis

Free Software Foundation’s R packages were used to compute descriptive and analytical statistical analyses. Sample size calculation was based on our primary endpoint using Obuchowsky’s method [24], and 34 patients were needed to detect an area under the curve of the receiver operating characteristic (ROC) curve of 0.8 with a power of 0.9 and an alpha risk of 0.05. The ratio between CRT-R and CRT-NR in our population was hypothesised to be 0.5. A Shapiro-Wilk test was used to test the normal distribution of the data. Data were expressed as mean ± standard deviation or median [25th–75th interquartile range (IQR)] according to their distribution. The inter- and intra-observer reproducibility for CRT measurements were evaluated by the coefficient of variation. The definition of CRT-R was a CRT reduction of at least 25%. This was based on the least significant change (LSC) of the CRT of previous non-published personal data and then challenged by the LSC of the CRT in this cohort. As the LSC arbitrarily defines this threshold, we also displayed the results for different thresholds to define CRT-R and CRT-NR (Additional file 1: Annex 1). Pairwise comparisons of data were done with the paired Student’s *t* test or Wilcoxon test. The two-tailed Student *t* test or Mann-Whitney *U* test compared CRT-R and CRT-NR. Fisher test and *χ*^2^ were used appropriately to compare categorical data. To compare the effect of the group (CRT-R/CRT-NR) and time (T1, T2, T3, T4) on haemodynamic variables, we used a linear mixed-effect model using time as a variable with a fixed effect, and patient and group as variables with a random effect for intercept and slopes, respectively. Visual inspection of residual plots assessed the absence of deviations from homoscedasticity or normality. Dunnett’s test enabled multiple comparisons to the baseline for each haemodynamic variable. CRT was expressed as a variation from baseline, computed as the difference between final and baseline value divided by the baseline value. Pearson’s correlation coefficient tested the linear correlations. ROC curves were built, and AUC was expressed with 95% confidence interval (CI) calculated with a bootstrap method using 2000 repetitions. Best thresholds were determined by the “closest top-left” method, and sensitivity, specificity, and positive and negative predictive values were expressed with 95% CI. The grey zone was determined with a two-step method: First, a bootstrap resampling method was applied on ΔCRT-PLR and basal CRT and PCO_2_gap data. The best threshold and its 95% CI were calculated for each variable using a bootstrap technique with 2000 repetitions to define a first inconclusive zone. Secondly, we determined the cut-off values with a sensitivity less than 90% or specificity less than 90% defining a second inconclusive zone. The larger of the two zones were retained as the grey zone. All the tests were two-sided, and a *p* value < 0.05 was considered statistically significant.

## Results

### Patient characteristics

**Fig. 1:**
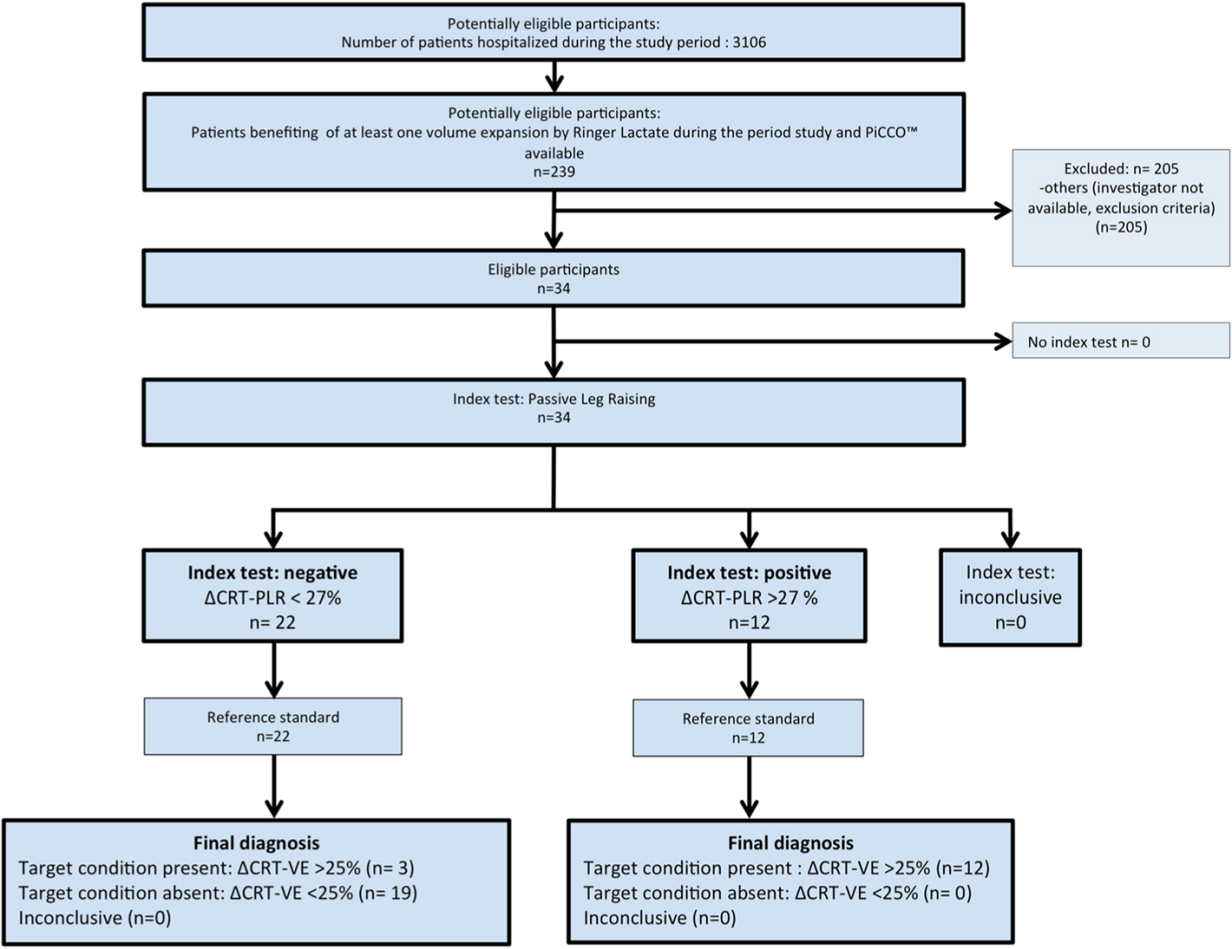
Flow chart of the study. CRT, capillary refill time; ∆CRT-PLR, capillary refill time variation induced by passive leg raising; ∆CRT-PLR > 27%, positive index test defined as a decrease of capillary refill time induced by passive leg raising of at least 27%; ∆CRT-VE > 25%, CRT response defined as a decrease of capillary refill time induced by volume expansion of at least 25%; PLR, passive leg raising; VE, volume expansion

We included 34 non-consecutive patients in the study period (Fig. 1). Fifteen (44%) patients were CRT-R, and 19 (56%) were CRT-NR. The main characteristics of the patients’ population are shown in Table 1. Ten patients died before day 90 (29.4%). We assessed CRT with 4 videos in each of the 3 steps of the study, except for 1 patient who had only 9 over 12 video acquisitions due to a technical issue; their data were included in the final analysis. Blood gases were missing in 6 patients due to transport and analytical issues. CRT and PCO_2_gap only were significantly different between CRT-R and CRT-NR (Table 1). Using a response based on the cardiac index (CI), and defined as an increase of at least 15% following VE, 13 (38%) patients were cardiac index responders (CI-R) while 21 (62%) were not (CI-NR). Comparing CI-R and CI-NR for the same characteristics and haemodynamic variables, we did not find any significant differences, except for CI, oxygen delivery, and CRT at baseline: 2.7 s [IQR, 2.3–2.9] in CI-NR vs. 3.7 s [IQR, 3.1–4.7] in CI-R (*p* = 0.018) (Additional file 1: Annex 5). We did not observe any adverse event from performing both CRT and PLR. Macrocirculatory, peripheral perfusion, and metabolic variables in CRT-R and CRT-NR at the different steps of the experimental protocol are shown in Table 2. Only CRT changed significantly during PLR and volume expansion in the CRT-R compared to the CRT-NR.

**Table 1:** Patients’ demographic and clinical characteristics in capillary refill time responders and non-responders to volume expansion

Characteristics	All (*n* = 34)	CRT responders (*n* = 15)	CRT non-responders (*n* = 19)	*p* value
Anthropometry				
Age, years	62 [54, 69]	59 [46, 71]	64 [57, 68]	0.435
Sex, male/female, *n*	25/9	13/2	12/7	
Weight, kg	71 [62, 80]	75 [67, 81]	70 [61, 78]	0.289
Severity scores				
SOFA	8 [6, 11]	9 [7, 12]	8 [6, 10]	0.485
SAPS2	42 [33, 51]	44 [33, 56]	41 [34, 48]	0.266
Circulatory failure aetiology				
Sepsis	11	3	8	0.271
SIRS	15	8	7	0.489
Cardiogenic shock	8	4	4	1.0
Metabolic and peripheral perfusion				
Mottling score	1 [0, 2]	1 [0, 2]	1 [0, 1]	0.702
Capillary refill time, s	2.9 [2.4, 3.7]	3.6 [2.8, 6]	2.6 [2.3, 3.3]	0.021
PCO_2_gap, kPa	1.1 [0.8, 1.7]	1.4 [1.1, 1.8]	0.8 [0.7, 1.1]	0.007
Oxygen delivery, mL min^−1^ m^−2^	337 [272, 417]	313 [253, 361]	355 [309, 440]	0.228
Oxygen uptake, mL min^−1^ m^−2^	101 [84, 116]	97 [64, 119]	103 [88, 109]	0.383
Modified RQ, mmHg mL^−1^	2.6 [2.0, 5.4]	3.5 [2.4, 6.8]	2.6 [2.0, 4.0]	0.118
Oxygen extraction ratio, %	30 [25, 35]	35 [26, 40]	28 [23, 30]	0.051
Lactate, mmol L^−1^	2.0 [1.2, 3.8]	2.0 [1.4, 3.8]	2.0 [1.1, 3.5]	0.578
Thermodilution				
Cardiac index, L min^−1^ m^−2^	2.6 [2.1, 3.1]	2.3 [2.0, 3.0]	2.6 [2.4, 3.2]	0.499
Global end-diastolic volume index, mL m^−2^	710 [549, 827]	667 [506, 826]	726 [606, 823]	0.492
Extravascular lung water index, mL kg^−1^	9.1 [7.0, 12.0]	9.2 [6.5, 12.5]	9.1 [7.2, 11.5]	0.931
Cardiac function index	3.6 [2.9, 4.3]	3.6 [2.8, 4.3]	3.6 [3.1, 4.3]	0.718
Pulmonary vascular permeability index	1.8 [1.4, 2.6]	1.8 [1.5, 2.7]	1.7 [1.3, 2.5]	0.772
Stroke volume variation, %	13 [9, 17]	14 [11, 17]	13 [8, 17]	0.676
Haemodynamics				
Heart rate, cycle/min	95 [75, 110]	96 [73, 104]	93 [78, 112]	0.395
Mean arterial pressure, mmHg	68 [61, 72]	70 [62, 75]	67 [61, 71]	0.357
Systolic arterial pressure, mmHg	102 [91, 118]	97 [90, 116]	104 [94, 116]	0.795
Diastolic arterial pressure, mmHg	51 [43, 57]	52 [46, 57]	51 [41, 56]	0.615
Pulse pressure, mmHg	53 [40, 65]	49 [36, 65]	53 [41, 64]	0.532
EtCO_2_, mmHg	36 [32, 45]	34 [32, 44]	37 [31, 46]	0.737
Central venous pressure, mmHg	7 [4, 11]	8 [7, 11]	6 [3, 9]	0.186
Drugs				
Norepinephrine, μg kg^−1^ min^−1^	0.22 [0.10, 0.51]	0.17 [0.08, 0.76]	0.23 [0.11, 0.49]	0.798
Dobutamine, μg kg^−1^ min^−1^	4 [0, 6]	4 [0, 5]	4 [0, 7]	0.531
Epinephrine, μg kg^−1^ min^−1^	0.00 [0.00, 0.04]	0.00 [0.00, 0.00]	0.00 [0.00, 0.05]	0.472
Ventilation				
Tidal volume, mL kg^−1^ of ideal body weight	6 [6, 7]	6 [6, 7]	6 [6, 8]	0.702
Respiratory rate, cycles min^−1^	24 [20, 27]	24 [23, 27]	22 [17, 29]	0.357
Positive end-expiratory pressure, cmH_2_O	5 [5, 6]	6 [5, 6]	5 [5 6]	0.937
FiO_2_, %	40 [30, 60]	40 [36, 51]	40 [30, 60]	0.607
Driving pressure, cmH_2_O	12 [9, 15]	12 [10, 14]	12 [9, 15]	0.806

Values are median [percentile 25–75] or number. Wilcoxon test and Fisher’s exact test were used to calculate *p* value. CRT responders are defined as patients showing a decrease of at least 25% of the capillary refill time after volume expansion

*CRT* capillary refill time, *EtCO*_*2*_ end-tidal carbon dioxide, *FiO*_*2*_ fraction of inspired dioxygen, *Modified RQ* modified respiratory quotient defined as PCO_2_gap/difference in arterio-venous content in oxygen, *PCO*_*2*_*gap* veno-arterial difference in carbon dioxide partial pressure, *SAPS2* Simplified Acute Physiology Score, *SIRS* systemic inflammatory response syndrome, *SOFA* Sequential Organ Failure Assessment

**Table 2:** Changes in haemodynamic (A) and metabolic (B) parameters over the study period

A								
		Baseline (T1)	During PLR (T2)	Baseline (T3)	After VE (T4)	Group R/NR	Study period	R/NRstudy period
CRT, s	CRT-R (*n* = 15)	3.6 [2.8, 6.0]	2.3 [1.6, 3.6]	NA	2.1 [1.8, 2.9]*	< 0.001	0.972	< 0.001
	CRT-NR (*n* = 19)	2.6 [2.3, 3.3]	2.6 [2.0, 3.1]	NA	2.6 [2.0, 3.1]			
CI, L min^−1^ m^−2^	CRT-R (*n* = 15)	2.5 [2.0, 3.1]	2.8 [2.5, 3.3]	2.5 [2.1, 3.2]	3.0 [2.7, 3.2]	0.987	< 0.001	0.829
	CRT-NR (*n* = 19)	2.5 [2.2, 3.1]	2.8 [2.5, 3.5]	2.4 [2.1, 3.3]	3.1 [2.6, 3.4]			
SVi, mL m^−2^	CRT-R (*n* = 15)	31 [24, 35]	34 [27, 37]	30 [24, 36]	33 [30, 40]	0.787	0.001	0.997
	CRT-NR (*n* = 19)	27 [22, 35]	31 [30, 37]	27 [21, 36]	32 [27, 36]			
HR, min^−1^	CRT-R (*n* = 15)	96 [73.0, 104]	97 [72, 105]	96 [73, 106]	93 [70, 105]*	0.700	0.674	0.537
	CRT-NR (*n* = 19)	93 [78, 112]	94 [78, 110]	95 [79, 110]	91 [80, 108]			
SAP, mmHg	CRT-R (*n* = 15)	97 [90, 116]	116 [102, 136]	107 [94, 132.]	118 [105, 136]	0.624	0.477	0.554
	CRT-NR (*n* = 19)	104 [94, 116]	112 [99, 116]	98 [94, 110]	112 [104, 119]			
DAP, mmHg	CRT-R (*n* = 15)	52 [46, 57]	53 [46, 64]	49 [43, 54]	57 [45, 66]	0.958	0.524	0.249
	CRT-NR (*n* = 19)	51 [41, 56]	51 [47, 56]	51 [46, 54]	51 [46, 57]			
MAP, mmHg	CRT-R (*n* = 15)	70 [62, 75.]	71 [65, 82]	68 [60, 73]	76 [69, 81]	0.714	0.101	0.932
	CRT-NR (*n* = 19)	67 [61, 70]	67 [63, 76.]	64 [58, 70]	68. [63, 75]			
PP, mmHg	CRT-R (*n* = 15)	49 [36, 65.]	55 [46, 83]	58 [44, 83]	60 [46, 75]	0.321	0.051	0.548
	CRT-NR (*n* = 19)	53 [41, 64]	56 [49, 63]	51 [44, 58]	58 [50, 72]			
SpO_2_, %	CRT-R (*n* = 15)	98 [97, 100]	99 [97, 100]	99 [97, 100]	100 [97, 100]	0.353	0.143	0.575
	CRT-NR (*n* = 19)	99 [95,100]	98 [95, 100]	97 [93, 100]	99 [96, 100]			
EtCO_2_, mmHg	CRT-R (*n* = 15)	34 [32, 44]	36 [32, 45]	34 [30, 43]	34 [29.7, 45.5]	0.708	0.175	0.548
	CRT-NR (*n* = 19)	37 [31, 46]	36 [32, 44]	35 [30, 43]	36 [31, 43]			
CVP, mmHg	CRT-R (*n* = 15)	8 [7, 11]	11 [9, 14]	8 [6, 11]	10 [7, 13]	0.678	0.906	0.543
	CRT-NR (*n* = 19)	6 [3, 9]	7 [5, 12]	5 [2, 8]	6 [4, 9]			
B								
		Baseline (T1)			After VE (T4)			*p* value
Oxygen delivery, mL min^−1^ m^−2^	CRT-R (*n* = 14)	313 [254–361]			370 [317–399]			0.002
	CRT-NR (*n* = 14)	355 [309–440]			397 [327–458]			0.003
	*p* value	0.24			0.697			
Oxygen uptake, mL min^−1^ m^−2^	CRT-R (*n* = 14)	103 [88–109]			100 [90–109]			0.357
	CRT-NR (*n* = 14)	97 [64–119]			104 [80–133]			0.375
	*p* value	0.401			0.697			
Oxygen extraction ratio	CRT-R (*n* = 14)	0.34 [0.26–0.40]			0.28 [0.22–0.33]			0.024
	CRT-NR (*n* = 14)	0.28 [0.23–0.30]			0.24 [0.19–0.33]			0.583
	*p* value	0.051			0.608			

Values are median [IQR]. A mixed-effect linear model was used to compute p value. Dunnett’s test was performed for multiple comparisons to the baseline

*p < 0.05

*CRT* capillary refill time, *CI* cardiac index, *CVP* central venous pressure, *DAP* diastolic arterial pressure, *EtCO*_*2*_ end-tidal carbon dioxide, *HR* heart rate, *IQR* 25th–75th interquartile range, *MAP* mean arterial pressure, *CRT-R* responders to volume expansion defined as patients showing a decrease in CRT of at least 25% after volume expansion, *CRT-NR* non-responders to volume expansion define as patients showing a decrease in CRT after VE of less than 25%, *PLR* passive leg raising, *PP* pulse pressure, *SAP* systolic arterial pressure, *DAP* diastolic arterial pressure, *SpO*_*2*_ dioxygen pulse saturation, *SVi* stroke volume index, *VE* volume expansion

**Fig. 2:**
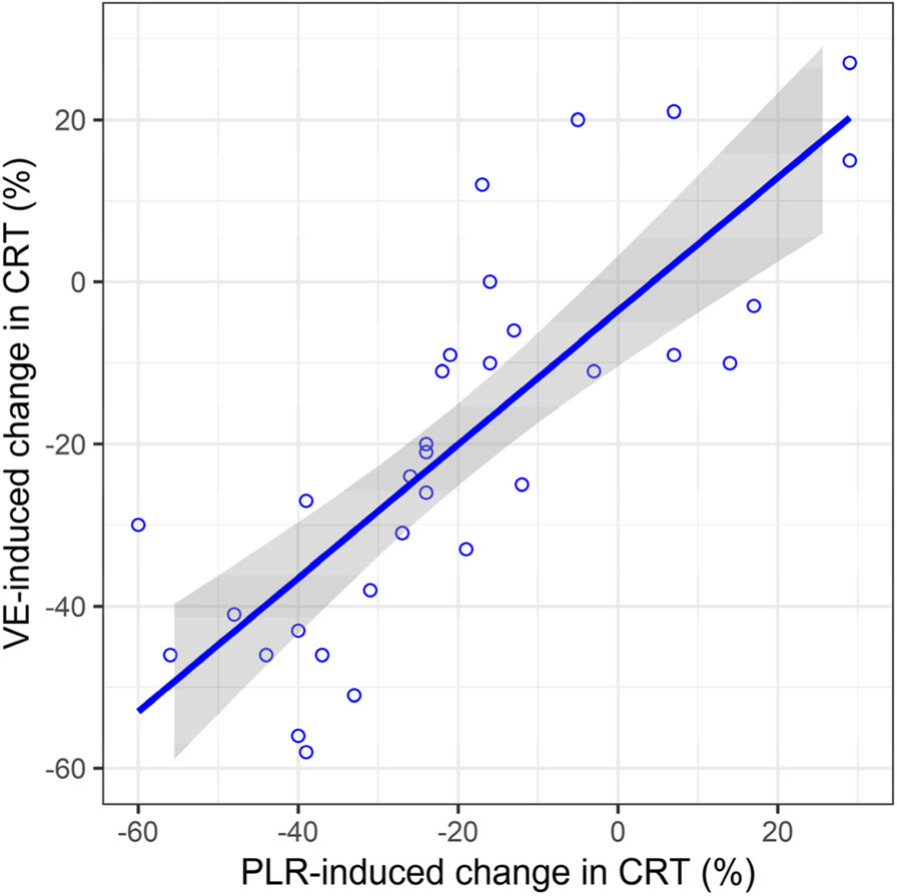
Scatter plot of capillary refill time variation induced by passive leg raising vs. by volume expansion. CRT, capillary refill time; PLR, passive leg raising; VE, volume expansion

The relationship between ΔCRT-PLR and ΔCRT after volume expansion is depicted in Fig. 2 (*r*^2^ = 0.62; *p* < 0.001). Individual CRT in CRT-R and CRT-NR, at baseline, during PLR, and after volume expansion are depicted in Additional file 1: Annex 4. We did not find any significant correlation between the changes in CRT and changes in macrocirculatory and metabolic variables induced by VE. However, the PCO_2_gap was higher in CRT-R and the oxygen extraction ratio was almost significantly higher in CRT-R at baseline and decreased significantly only in CRT-R following volume expansion. Median CRT at baseline was 2.7 s [IQR, 2.4–3.5] in survivors and 4.4 s [IQR, 3.1–6.3] in non-survivors (*p* = 0.021).

### Relationship between CRT responders and CI responders to volume expansion

Twenty-one patients were CI-NR, and 13 were CI-R. Seven patients (54%) were CRT-R in the 13 CI-R. Thirteen patients (61%) were CRT-NR in the 21 CI-NR patients, and the odds ratio was 1.86 [95% CI, 0.38; 9.63]. Among the 19 patients with a CRT of less than 3 s, only 3 were CI-R. Among the 15 patients with a CRT of more than 3 s, 10 were CI-R and 9 were CRT-R.

### Reproducibility measurements

In 11 patients, 10 consecutive CRT were collected to assess the intra-observer variability. The coefficient of variation for intra-observer variability was 17.6% [95% CI, 14.4–20.9] for a single CRT measurement and 8.8% [95% CI, 6.7–11.0] for 4 CRT measurements. The LSC for 4 measurements was 25.0% [95% CI, 17.7–29.6]. In 20 patients, CRT was analysed 4 times by 2 observers (MJL and NB). The coefficient of variation for inter-observer variability was 7.3% [95% CI, 3.9–10.2].

### Prediction of CRT responsiveness

A 27% decrease in CRT-PLR predicted CRT responsiveness with a sensitivity of 87% [95% CI, 73–100] and a specificity of 100% [95% CI, 74–100]. The ROC_AUC_ of ΔCRT-PLR was 0.94 [95% CI, 0.87–1.0]. Using the grey zone approach, inconclusive values ranged from − 30 to − 18% for ΔCRT-PLR to predict CRT responsiveness, including 24% of the patients. Using different thresholds to define CRT responsiveness, namely 15%, 20%, 30%, and 35%, ROC_AUC_ were 0.97 [95% CI, 0.93–1.00], 0.97 [95% CI, 0.92–1.00], 0.96 [95% CI, 0.88–1.00], and 0.94 [95% CI, 0.84–1.00], respectively (Additional file 1: Annex 1).

**Fig. 3:**
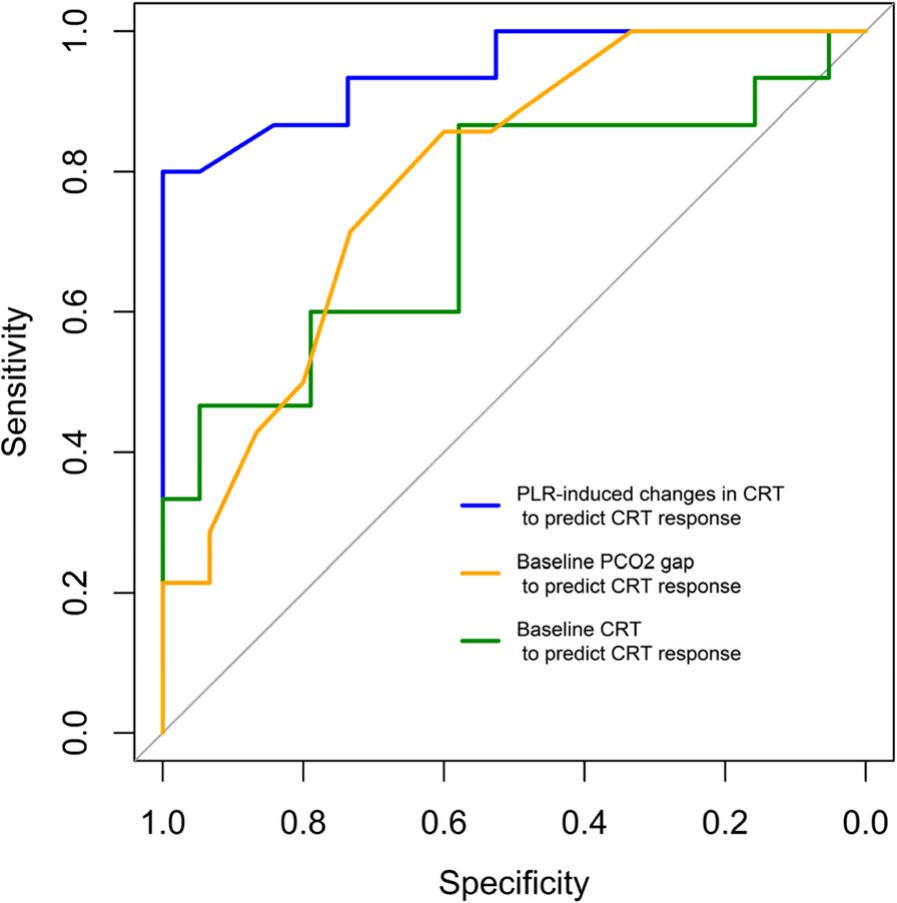
ROC curves of CRT and ∆CRT-PLR to predict CRT response to volume expansion. CRT, capillary refill time; CRT responders, response to volume expansion defined as patients showing a decrease in CRT after VE of at least 25%; PCO_2_gap, central venous-to-arterial carbon dioxide difference; PLR, passive leg raising; VE, volume expansion

Two other variables predicted CRT responsiveness: baseline CRT with a ROC_AUC_ of 0.73 [95% CI, 0.54–0.90] and PCO_2_gap with a ROC_AUC_ of 0.79 [95% CI, 0.61–0.93]. Using the grey zone approach, inconclusive values ranged from 2.6 to 4.1 s for baseline CRT (including 44% of the patients) and from 0.9 to 1.7 kPa for PCO_2_gap (including 32% of the patients). Comparative abilities of ΔCRT-PLR, baseline CRT, and PCO_2_gap to predict CRT responsiveness are shown in Fig. 3 and Table 3.

## Discussion

The main results are as follows: (1) changes in CRT during PLR predicts CRT responsiveness with a good accuracy in acute circulatory failure, and the best threshold to assess CRT responsiveness is a CRT decrease by 27% during PLR; (2) baseline CRT and PCO_2_gap are also able to predict CRT responsiveness; (3) baseline CRT is longer in non-survivors than in survivors; and (4) peripheral perfusion, macrocirculatory, and metabolic variables are poorly correlated. The originality of the present study was to investigate a method predicting the effect of volume expansion on peripheral perfusion by using a simple, non-invasive, costless, static, and dynamic clinical sign, namely the CRT.

To carry out this work, we used a rigorous approach. First, to increase both precision and reproducibility of the CRT, we used a piston to calibrate the compression and we recorded the CRT on a video with controlled luminosity [23], enabling a blind lecture. Second, we averaged four measurements for each haemodynamic condition. Thus, we minimised the inter- and intra-observer variabilities compared with previous reports [24]. As we studied the variation in the same patients, we controlled other variables which might have influenced the CRT values, such as ambient temperature [25] and inter-patient variability linked to age or sex [24]. Third, to assess the precision of our measurement, we calculated the LSC of CRT. It helped us to define a threshold to differentiate random variation due to fluctuation in the measurement and real change of the CRT that defined CRT responsiveness. The same method was used to define a fluid responder regarding cardiac output [26]. The assessment of cardiac index by transpulmonary thermodilution was performed as recommended. This technique provides a LSC of 12% [26], which is below our definition of cardiac index response. CRT values were significantly longer in patients dying in an intensive care unit than in survivors, as previously described [12]. Finally, diagnostic accuracy studies are at high risk of biases [27], but we minimised them by applying STARD guidelines [19] and reporting our adhesion to nearly all items (Additional file 1: Annex 6).

Improvement of CRT by using volume expansion was reported [28], and a dissociation between macrocirculation and microcirculation or peripheral perfusion is well known [4, 29]. The dissociation may also be due to the lack of precision of both techniques, as the LSC of transpulmonary thermodilution cardiac index was up to 17% [26] (Table 3). Preload modifications, as volume expansion and PLR, can lead to changes in cardiac output [16], but also in arterial pressure according to patients’ dynamic elastance [30], and in central venous pressure and blood viscosity [31]. A reduction of sympathetic tone can increase microvascular flow [18]. The significant decrease in heart rate in CRT-R in the current study supports that idea. Though preload modification may change arterial pressure, venous pressure, sympathetic tone, and fluid administration can change blood viscosity. All those changes may have an opposing effect on microcirculation and peripheral perfusion. An approach based directly on peripheral perfusion such as the CRT could be interesting in that context. Initial CRT predict the reduction of CRT after volume expansion, and this finding is consistent with the microvascular flow index findings, analysed with videomicroscopy, where an initial low microvascular flow index predicts an increase of microvascular flow index after volume expansion [15]. The absence of correlation with metabolic variables could be explained by the lack of precision of the biological tests [32]. The inherent natural variability of PaO_2_ is important [17]. This could explain the inability of oxygen uptake, oxygen delivery, and the modified respiratory quotient [33] to be linked with CRT responsiveness in our study. Contrariwise, PCO_2_gap has less variability.

**Table 3:** Diagnostic performances to predict CRT and cardiac index responsiveness

	Index test	AUC [95% CI]	Best threshold	Specificity	Sensitivity	PPV	NPV	Youden index
Peripheral perfusion response (decrease of 25% of CRT after VE)	ΔCRT-PLR	0.94 [0.87–1.0]	− 27% [− 30, − 18]	1.00 [0.74–1.00]	0.87 [0.73–1.0]	1.0 [0.72–1.0]	0.91 [0.79–1.0]	0.87
	PCO_2_gap	0.79 [0.61–0.93]	1.2 kPa [0.9–1.6]	0.73 [0.53–0.93]	0.79 [0.57–1.00]	0.72 [0.58–0.92]	0.79 [0.62–1.00]	0.52
	Baseline CRT	0.73 [0.54–0.90]	2.7 s [2.6–4.1]	0.74 [0.47–1.0]	0.80 [0.47–1.00]	0.69 [0.54–1.00]	0.67 [0.8–1.00]	0.49
Cardiac index response (increase of 15% of CI)	ΔCI-PLR	0.95 [0.86–1.00]	9.0% [8.7–13.7]	0.90 [0.71–1.00]	1.00 [0.77–1.00]	0.85 [0.68–1.00]	1.00 [0.86–1]	0.90

*ROC*_*AUC*_ area under the receiving operating characteristic curves, *CRT* capillary refill time, *VE* volume expansion, *ΔCRT-PLR* variation of CRT induced by passive leg raising (PLR), *ΔCI-PLR* variation of cardiac index induced by PLR, *NPV* negative predictive value, *PPV* positive predictive value

We did not expect to have that much CRT-R patients among CI-NR patients. In peripheral perfusion-targeted therapy, only the patient showing fluid responsiveness on CI had a fluid load, and this strategy was stopped when peripheral perfusion targets were obtained [15]. We are not sure that patients who are CRT-R and CI-NR would benefit from a volume expansion, and those results should be interpreted cautiously. Noteworthy, the median value of CRT before volume expansion in CI-NR is normal (less than 3 s) [15] and significantly lower than in CI-R. A restrictive approach on volume expansion that select patient with CRT of more than 3 s and that decrease significantly CRT during PLR could reasonably be tested and may reduce further fluid overload [14].

We acknowledge that our method to assess CRT is time-consuming and may be unrealistic in routine clinical practice. Future developments to digitalise CRT, providing real-time measurements, could facilitate its routine use [37].

Our study contains limitations. First, we did not have a gold standard to define microcirculation improvement. Such a standard does not presently exist, and each method explores a single window of the microvascular bed [34]. A way to validate the relevance of a microcirculation assessment technique is to check the link with outcomes and mortality [35]. This has been done with CRT [12]. Second, the studied population is heterogeneous with a majority of surgical patients experiencing a systemic inflammatory response syndrome due to cardiopulmonary bypass. This model of acute circulatory failure is not so far from the sepsis model, including vasoplegia, capillary leak, and contractility. Third, we are not sure that improvement of peripheral perfusion leads to less organ dysfunction and improved survival, as different microcirculatory beds may behave differently [36]. Goal-directed therapy protocols based on capillary refill time assessment have been tested and tend to be superior to protocols based on lactate assessment [15]. In this context, studies confirming our diagnostic method or other approaches predicting the effect of volume expansion on capillary refill time will be required. Fourth, the effect of PLR and volume expansion may have a different effect on tissue perfusion as blood viscosity evolution during PLR and VE was not the same and may alter the prediction accuracy of our method. Fifth, the sample size is quite small, and even if the study is positive for the primary endpoints, it impedes the valid interpretation of the association with other haemodynamic variables.

## Conclusion

We report above an original method to predict the effect of volume expansion on peripheral tissue perfusion, based on CRT measurement coupled to a passive leg raising manoeuvre. The method was accurate to predict the improvement in peripheral tissue perfusion of volume expansion. This method could be implemented in peripheral perfusion-targeted therapy leading to a restrictive fluid therapy approach. To be clinically feasible, this strategy needs to be confirmed using devices to assess an accurate, real-time digitalised CRT.

## Additional file

Additional file 1: Annex 1. AUC ROC according to the different thresholds to define CRT responders. Annex 2. Presentation of the piston to perform a calibrated compression of the skin before analysing the capillary refill time. Annex 3. Formulae used. Annex 4. Capillary refill time at the different time courses of the study in CRT responders and non-responders. Annex 5. Patients’ demographic and clinical characteristics in cardiac index responders and non-responders to volume expansion. Annex 6. Adhesion of our study to the STARD statement. (DOCX 923 kb)

## Data Availability

The datasets used and analysed during the current study are available from the corresponding author on reasonable request.

## Abbreviations

ANSM: Agence National de Sécurité du Médicament et des Produits de Santé
CI: Cardiac index
CI: Confidence interval
CI-NR: Cardiac index non-responders
CI-R: Cardiac index responders
CPP: Comité de Protection des Personnes
CRT: Capillary refill time
CRT-NR: Capillary refill time non-responders
CRT-R: Capillary refill time responders
ESICM: European Society of Intensive Care Medicine
IQR: Interquartile range
LSC: Least significant change
PCO_2_gap: Venous-to-arterial carbon dioxide tension difference
PLR: Passive leg raising
ROC_AUC_: Receiver operating characteristic curves were built and areas under the curves
SAPS2: Simplified acute physiology score
STARD: Standards for Reporting Diagnostic Accuracy
ΔCRT-PLR: Variation of CRT following passive leg raising
ΔCRT-VE: Variation of CRT following volume expansion

## References

[1] Cecconi Maurizio, De Backer Daniel, Antonelli Massimo, Beale Richard, Bakker Jan, Hofer Christoph, Jaeschke Roman, Mebazaa Alexandre, Pinsky Michael R., Teboul Jean Louis, Vincent Jean Louis, Rhodes Andrew (2014). Consensus on circulatory shock and hemodynamic monitoring. Task force of the European Society of Intensive Care Medicine. Intensive Care Medicine.

[2] Rhodes A, Evans LE, Alhazzani W, Levy MM, Antonelli M, Ferrer R, et al. Surviving Sepsis Campaign: international guidelines for management of sepsis and septic shock. Crit Care Med. 2017;1. 10.1097/CCM.0000000000005337 34605781

[3] De Backer D, Creteur J, Preiser J, Dubois M, Vincent J (2002). Microvascular blood flow is altered in patients with sepsis. Am J Respir Crit Care Med.

[4] Garcia-Alvarez M, Marik P, Bellomo R (2014). Sepsis-associated hyperlactatemia. Crit Care.

[5] Brunauer A, Koköfer A, Bataar O, Gradwohl-Matis I, Dankl D, Bakker J (2016). Changes in peripheral perfusion relate to visceral organ perfusion in early septic shock: a pilot study. J Crit Care.

[6] Pickard A, Karlen W, Ansermino JM (2011). Capillary refill time: is it still a useful clinical sign?. Anesth Analg.

[7] Ait-Oufella H., Bige N., Boelle P. Y., Pichereau C., Alves M., Bertinchamp R., Baudel J. L., Galbois A., Maury E., Guidet B. (2014). Capillary refill time exploration during septic shock. Intensive Care Medicine.

[8] Fleming Susannah, Gill Peter, Jones Caroline, Taylor James A., Van den Bruel Ann, Heneghan Carl, Roberts Nia, Thompson Matthew (2015). The Diagnostic Value of Capillary Refill Time for Detecting Serious Illness in Children: A Systematic Review and Meta-Analysis. PLOS ONE.

[9] Hernandez G, Pedreros C, Veas E, Bruhn A, Romero C, Rovegno M (2012). Evolution of peripheral vs metabolic perfusion parameters during septic shock resuscitation. A clinical-physiologic study. J Crit Care.

[10] de Moura Edmilson Bastos, Amorim Fábio Ferreira, da Cruz Santana Alfredo Nicodemos, Kanhouche Gabriel, de Souza Godoy Lucas Garcia, de Jesus Almeida Lucila, Rodrigues Thais Almeida, da Silveira Carlos Darwin Gomes, de Oliveira Maia Marcelo (2016). Skin mottling score as a predictor of 28-day mortality in patients with septic shock. Intensive Care Medicine.

[11] Ait-Oufella H, Lemoinne S, Boelle PY, Galbois A, Baudel JL, Lemant J (2011). Mottling score predicts survival in septic shock. Intensive Care Med.

[12] Hernandez G, Bruhn A, Castro R, Regueira T. The holistic view on perfusion monitoring in septic shock. Curr Opin Crit Care. 2012. 10.1097/MCC.0b013e3283532c08 22473257

[13] Dünser MW, Takala J, Brunauer A, Bakker J (2013). Re-thinking resuscitation: leaving blood pressure cosmetics behind and moving forward to permissive hypotension and a tissue perfusion-based approach. Crit Care.

[14] van Genderen ME, Engels N, van der Valk RJP, Lima A, Klijn E, Bakker J (2015). Early peripheral perfusion-guided fluid therapy in patients with septic shock. Am J Respir Crit Care Med.

[15] Hernández G, Ospina-Tascón GA, Damiani LP, Estenssoro E, Dubin A, Hurtado J (2019). Effect of a resuscitation strategy targeting peripheral perfusion status vs serum lactate levels on 28-day mortality among patients with septic shock: the ANDROMEDA-SHOCK randomized clinical trial. JAMA..

[16] Pranskunas Andrius, Koopmans Matty, Koetsier Peter M., Pilvinis Vidas, Boerma E. Christiaan (2012). Microcirculatory blood flow as a tool to select ICU patients eligible for fluid therapy. Intensive Care Medicine.

[17] Monnet X, Marik P, Teboul J-L. Passive leg raising for predicting fluid responsiveness: a systematic review and meta-analysis. Intensive Care Med. 2016. 10.1007/s00134-015-4134-1 26825952

[18] Pottecher J, Deruddre S, Teboul J-L, Georger J-F, Laplace C, Benhamou D (2010). Both passive leg raising and intravascular volume expansion improve sublingual microcirculatory perfusion in severe sepsis and septic shock patients. Intensive Care Med.

[19] Pettilä V, Merz T, Wilkman E, Perner A, Karlsson S, Lange T (2016). Targeted tissue perfusion versus macrocirculation-guided standard care in patients with septic shock (TARTARE-2S): study protocol and statistical analysis plan for a randomized controlled trial. Trials..

[20] Bossuyt PM, Reitsma JB, Bruns DE, Gatsonis CA, Glasziou PP, Irwig L, et al. STARD 2015: an updated list of essential items for reporting diagnostic accuracy studies. BMJ. 2015:h5527. 10.1136/bmj.h5527 PMC4623764 26511519

[21] Vincent JL, Moreno R, Takala J, Willatts S, De Mendonça A, Bruining H (1996). The SOFA (Sepsis-related Organ Failure Assessment) score to describe organ dysfunction/failure. On behalf of the Working Group on Sepsis-Related Problems of the European Society of Intensive Care Medicine. Intensive Care Med.

[22] Le Gall JR, Lemeshow S, Saulnier F (1993). A new Simplified Acute Physiology Score (SAPS II) based on a European/North American multicenter study. JAMA..

[23] Monnet X, Teboul J-L (2015). Passive leg raising: five rules, not a drop of fluid!. Crit Care Lond Engl.

[24] Obuchowski NA (1998). Sample size calculations in studies of test accuracy. Stat Methods Med Res.

[25] Brown LH, Prasad NH, Whitley TW (1994). Adverse lighting condition effects on the assessment of capillary refill. Am J Emerg Med.

[26] Anderson B, Kelly A-M, Kerr D, Clooney M, Jolley D (2008). Impact of patient and environmental factors on capillary refill time in adults. Am J Emerg Med.

[27] Schriger DL, Baraff L (1988). Defining normal capillary refill: variation with age, sex, and temperature. Ann Emerg Med.

[28] Monnet Xavier, Persichini Romain, Ktari Mariem, Jozwiak Mathieu, Richard Christian, Teboul Jean-Louis (2011). Precision of the transpulmonary thermodilution measurements. Critical Care.

[29] Jacquet-Lagrèze M, Izaute G, Fellahi J-L (2017). Diagnostic accuracy studies: the methodologic approach matters!. Anesthesiology..

[30] Maitland K, Pamba A, Newton CRJC, Levin M (2003). Response to volume resuscitation in children with severe malaria. Pediatr Crit Care Med.

[31] De Backer D, Ortiz JA, Salgado D. Coupling microcirculation to systemic hemodynamics. Curr Opin Crit Care. 2010;16:250–4. 10.1097/MCC.0b013e3283383621 20179590

[32] De Backer D, Donadello K, Sakr Y, Ospina-Tascon G, Salgado D, Scolletta S, et al. Microcirculatory alterations in patients with severe sepsis: impact of time of assessment and relationship with outcome. Crit Care Med. 2013;41:791–9. 10.1097/CCM.0b013e3182742e8b 23318492

[33] García MIM, Romero MG, Cano AG, Aya HD, Rhodes A, Grounds RM, et al. Dynamic arterial elastance as a predictor of arterial pressure response to fluid administration: a validation study. Crit Care. 2014;18:626. 10.1186/s13054-014-0626-6 PMC4271484 25407570

[34] Guérin L, Teboul J-L, Persichini R, Dres M, Richard C, Monnet X. Effects of passive leg raising and volume expansion on mean systemic pressure and venous return in shock in humans. Crit Care. 2015;19. 10.1186/s13054-015-1115-2 PMC4657233 26597901

[35] Klijn Eva, Niehof Sjoerd, Johan Groeneveld A. B., Lima Alexandre Pinto, Bakker Jan, van Bommel Jasper (2015). Postural change in volunteers: sympathetic tone determines microvascular response to cardiac preload and output increases. Clinical Autonomic Research.

[36] Mallat J, Lazkani A, Lemyze M, Pepy F, Meddour M, Gasan G, et al. Repeatability of blood gas parameters, PCO2 gap, and PCO2 gap to arterial-to-venous oxygen content difference in critically ill adult patients. Medicine (Baltimore). 2015;94. 10.1097/MD.0000000000000415 PMC4602629 25621691

[37] Blaxter L L, Morris D E, Crowe J A, Henry C, Hill S, Sharkey D, Vyas H, Hayes-Gill B R (2015). An automated quasi-continuous capillary refill timing device. Physiological Measurement.

