# Supplementary material for: Capillary refill time variation induced by passive leg raising predicts capillary refill time response to volume expansion
Source: Crit Care. 2019 Aug 16;23:281. doi: 10.1186/s13054-019-2560-0 (PMC6697974; doi:10.1186/s13054-019-2560-0)
Supplement: Supplementary file 1 — Annex 1. AUC ROC according to the different thresholds to define CRT responders. Annex 2. Presentation of the piston to perform a calibrated compression of the skin before analysing the capillary refill time. Annex 3. Formulae used. Annex 4. Capillary refill time at the different time courses of the study in CRT responders and non-responders. Annex 5. Patients’ demographic and clinical characteristics in cardiac index responders and non-responders to volume expansion. Annex 6. Adhesion of our study to the STARD statement. (DOCX 923 kb) [file 13054_2019_2560_MOESM1_ESM.docx]

**Annex 1. AUC ROC according different thresholds to define CRT responders.**

| CRT response  (Decrease of CRT at different thresholds from 15 to 35 %) | AUC ROC | 95% Confidence interval |
| --- | --- | --- |
| 15% | 0.97 | [0.93-1.00] |
| 20% | 0.97 | [0.92- 1.00] |
| 25% | 0.94 | [0.87-1.0] |
| 30% | 0.96 | [0.88 -1.00] |
| 35% | 0.94 | [0.84-1.00] |

AUC ROC Area under the curve of the receiver operating curve; CRT Capillary refill time.

**Annex 2: Presentation of the piston to perform a calibrated compression of the skin before analysing the capillary refill time**

Image A shows the piston applied on the skin before skin compression. Image B shows the skin compression when 10 ml of air is compressed in a 7 ml volume to generate a pressure on the skin surface of 176 mm Hg on a 2.5 cm^2^ surface (personal data) during 7 seconds. Image C shows the skin surface capillary bed after pressure is applied to cause blanching.

**Annex 3. Formulae used**

Formulae used

CvO2, VO2, DO2, ER, RQ are approximate from true DO2, VO2, ER, RQ since venous blood from an upper vena cava central line were used instead of mixed venous blood sampling from a pulmonary arterial catheter. It was calculated as follow:

CaO2 = (Hb x 1.34 x SaO2) + (PaO2 x 0.003).

CvO2 = (Hb x 1.34 x ScvO2) + (PvO2 x 0.003).

VO2 = CO x (CaO2 - CvO2)

DO2 = CO x CaO2

ER = VO2/DO2.

PCO2gap= PvCO2-PaCO2

RQ modified =PCO2gap/ (CvO2- CaO2)

**Annex 4: Capillary refill time at the different time courses of the study in CRT responders and non-responders**


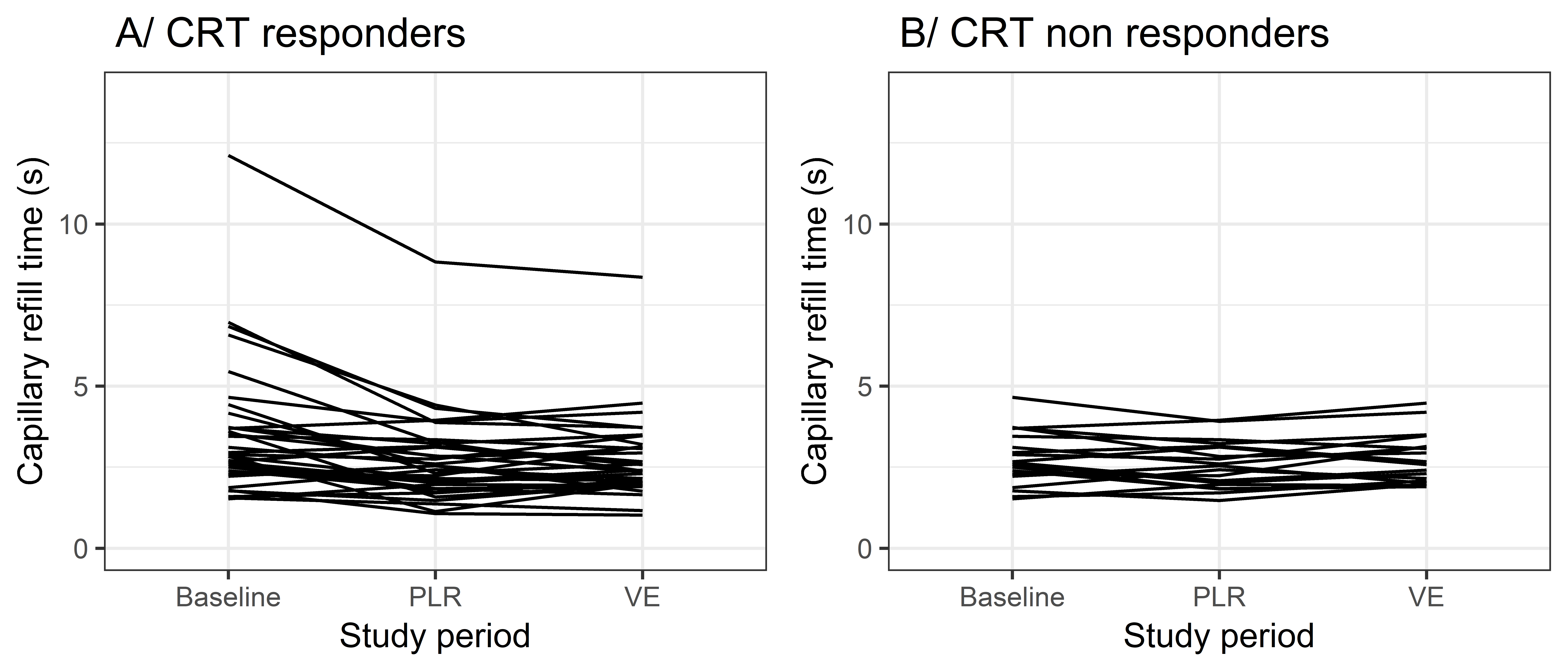


CRT responders: responders to volume expansion defined as patients showing a decrease in capillary refill time after VE of at least 25 %; CRT non-responders to volume expansion: define as patients showing a decrease in CRT after VE of less than 25 %; PLR: passive leg raising; VE: volume expansion.

**Annex 5. Patients demographic and clinical characteristics in cardiac index responders and non-responders to volume expansion**

|  |
| --- |

| **characteristics** | All (n=34) | CI-Non-responders (n=21) | CI-Responders (n=13) | p-value |
| --- | --- | --- | --- | --- |
| Age, year | 62 [54, 69] | 64 [54, 71] | 59 [48, 65] | 0.385 |
| Weight, kg | 71 [62, 80] | 73 [62, 80] | 70 [64, 82] | 0.887 |
| **Severity scores** |  |  |  |  |
| SOFA | 8 [6, 11] | 8 [5, 10] | 10 [8, 12] | 0.154 |
| SAPS2 | 42 [33, 51] | 41 [34, 50] | 44 [33, 51] | 0.582 |
| **Metabolic and peripheral perfusion** |  |  |  |  |
| Mottling score | 1 [0, 2] | 1 [0, 2] | 1 [1, 3] | 0.428 |
| Capillary refill time, s | 2.86 [2.42, 3.72] | 2.67 [2.29, 2.95] | 3.69 [3.11, 4.66] | 0.018 |
| Oxygen uptake, mL min^- 1^m^-2^ | 101 [84, 116] | 97 [75, 124] | 101 [84, 109] | 0.982 |
| Oxygen delivery, mL min^-1^ m^-2^ | 337 [272, 417] | 362 [335, 461] | 271 [206, 321] | 0.003 |
| PCO_2_ gap, kPa | 1.10 [0.80, 1.70] | 1.20 [0.80, 1.55] | 1.10 [0.80, 1.70] | 0.724 |
| Lactate, mmol L^-1^ | 1.98 [1.20, 3.82] | 1.88 [1.09, 3.16] | 2.20 [1.20, 5.00] | 0.357 |
| **Thermodilution** |  |  |  |  |
| Cardiac index, L min^-1^ m^-2^ | 2.60 [2.15, 3.08] | 3.00 [2.53, 3.35] | 2.04 [1.94, 2.32] | 0.002 |
| Extravascular lung water index mL kg^-1^ | 9 [7, 12] | 9 [8, 12] | 8 [6, 11] | 0.414 |
| Global end diastolic volume index, mL m^-2^ | 710 [549, 827] | 734 [589, 832] | 666 [531, 750] | 0.295 |
| Cardiac function index | 4 [3, 4] | 4 [3, 5] | 4 [3, 4] | 0.482 |
| Pulmonary vascular permeability index | 2 [1, 3] | 2 [2, 3] | 2 [1, 2] | 0.807 |
| **Haemodynamic** |  |  |  |  |
| Heart rate, cycle/min | 94 [75, 110] | 96 [75, 110] | 93 [75, 109] | 0.986 |
| Mean arterial pressure, mm Hg | 68 [60, 72] | 69 [62, 71] | 66 [60, 74] | 0.818 |
| Systolic arterial pressure, mm Hg | 102 [91, 118] | 104 [94, 112] | 94 [89, 124] | 0.547 |
| Diastolic arterial pressure, mm Hg | 51 [43, 57] | 51 [46, 57] | 51 [41, 55] | 0.804 |
| Pulse pressure, mm Hg | 53 [40, 65] | 52 [40, 62] | 55 [47, 66] | 0.831 |
| Central venous pressure, mm Hg | 7 [4, 11] | 7 [3, 8] | 10 [6, 12] | 0.194 |
| **Drugs** |  |  |  |  |
| Norepinephrine, µg kg^-1^min^-1^ | 0.22 [0.10, 0.51] | 0.18 [0.02, 0.45] | 0.31 [0.11, 0.85] | 0.145 |
| Dobutamine, µg kg^-1^min^-1^ | 4 [0, 6] | 4 [0, 6] | 5 [0, 10] | 0.367 |
| Epinephrine, µg kg^-1^min^-1^ | 0.00 [0.00, 0.04] | 0.00 [0.00, 0.00] | 0.00 [0.00, 0.10] | 0.222 |
| **Ventilation** |  |  |  |  |
| Tidal volume, mL kg^-1^ of ideal body weight | 6 [6, 7] | 6 [6, 7] | 7 [6, 8] | 0.702 |
| Driving pressure, cm H_2_O | 12 [9, 15] | 14 [9, 16] | 11 [9, 12] | 0.133 |
| positive end expiratory pressure, cm H_2_O | 5 [5, 6] | 5 [5, 7] | 5 [5, 6] | 0.479 |
| FiO_2_, | 0.40 [0.30, 0.60] | 0.40 [0.30, 0.60] | 0.40 [0.36, 0.51] | 0.607 |
| respiratory rate, cycles min^-1^ | 24 [20, 27] | 23 [18, 25] | 25 [22, 30] | 0.393 |
| EtCO2, mmHg | 36 [32, 46] | 34 [32, 41] | 42 [32, 53] | 0.269 |

**Annex 6: Adhesion of our study to the STARD statement.**

| SECTION: | Items, definition STARD |  |
| --- | --- | --- |
| TITLE OR ABSTRACT |  |  |
|  | 1 Identification as a study of diagnostic accuracy using at least one measure of accuracy (such as sensitivity, specificity, predictive values, or AUC) | Item reported:“ΔCRT-PLR predicted CRT responsiveness with a **sensitivity** of 87% [95% CI, 73-100] and a **specificity** of 100 % [95% CI, 74-100]. The **ROC_AUC_** of ΔCRT-PLR was 0.94 [95% CI, 0.87-1.0].” |
| ABSTRACT |  |  |
| reproducibility | 2 Structured summary of study design, methods, results, and conclusions (for specific guidance, see STARD for Abstracts) | Item reported |
| inter observer |  |  |
|  | 3 Scientific and clinical background, including the intended use and clinical role of the index test | Item reported: « A fluid challenge is recommended facing the association of macrocirculatory abnormalities and signs of tissue hypoperfusion including skin abnormalities, such as an increased CRT.[1]^,^[2] Many studies focused on the prediction of macrocirculatory effects of a volume expansion (VE) whereas those predicting the effects of VE on tissue perfusion are scarce” |
|  | 4 Study objectives and hypotheses | Item reported: “Therefore, we hypothesized that a rigorous protocol to measure CRT in association with PLR would be discriminant to predict CRT responsiveness in patients with circulatory shock.” |
| METHODS |  |  |
| Study design | 5 Whether data collection was planned before the index test and reference standard were performed (prospective study) or after (retrospective study) | Item reported « This prospective observational study was conducted in a 20-bed adult cardiothoracic intensive care unit in a tertiary teaching hospital (Louis Pradel hospital) in Lyon between September 2014 and December 2016 » |
| Participants | 6 Eligibility criteria | Item reported: “Eligibility criteria were as follows: The patient had to be equipped with an arterial and a central venous catheter, CRT had to be measurable, a CO monitoring by transpulmonary thermodilution (PiCCO™ PULSION Medical Systems, Munich, Germany) had to be implemented, and a 500 ml VE had to be prescribed by the attending intensivist. Circulatory failure was defined according to the ESICM guidelines.[1] We excluded patients with the following characteristics: pregnancy, cardiogenic pulmonary oedema, mechanical circulatory support, moribund patient, intra-abdominal hypertension, and lower limb amputation or an indication of compression stockings.” |
|  | 7 On what basis potentially eligible participants were identified (such as symptoms, results from previous tests, inclusion in registry) | Item reported by symptoms: ”Circulatory failure was defined according to the ESICM guidelines” |
|  | 8 Where and when potentially eligible participants were identified (setting, location and dates) | Item reported: “This prospective observational study was conducted in a 20-bed adult cardiothoracic intensive care unit in a tertiary teaching hospital (Louis Pradel hospital) in Lyon between September 2014 and December 2016.” |
|  | 9 Whether participants formed a consecutive, random or convenience series | Item reported: |
| Test methods | 10a Index test, in sufficient detail to allow replication | Item reported |
|  | 10b Reference standard, in sufficient detail to allow replication | Item reported |
|  | 11 Rationale for choosing the reference standard (if alternatives exist) | Item reported. “we don’t have a gold standard to define microcirculation improvement, but such a standard does not exist and each method only explore a particular window of the microvascular bed [35]. A way to validate the relevance of a microcirculation assessment technic is to check the link with mortality[36]. This has been done with CRT[12].” |
|  | 12a Definition of and rationale for test positivity cut-offs or result categories of the index test, distinguishing pre-specified from exploratory | Item reported: ”Best thresholds were determined by the “closest top left” method and sensitivity, specificity, positive and negative predictive value were expressed with 95 % confidence interval.” |
|  | 12b Definition of and rationale for test positivity cut-offs or result categories of the reference standard, distinguishing pre-specified from exploratory | Item reported: ”The definition of micro-R as a CRT reduction of at least 25% after VE was then challenged by the calculation of the least significant change (LSC) of the CRT. As this threshold is arbitrarily defined by the LSC, we also displayed results for different thresholds to define micro-R and micro-NR.” And “for 4 CRT measurements. The LSC for 4 measurements was 25.0% [95% CI: 17.7-29.6]” |
|  | 13a Whether clinical information and reference standard results were available to the performers/readers of the index test | Item reported“The readers were blinded to the clinical condition of patients and of the evaluation of the index test (ΔCRT-PLR) and the reference standard (ΔCRT-VE)” |
|  | 13b Whether clinical information and index test results were available to the assessors of the reference standard | Item reported: “The readers were blinded to the clinical condition of patients and of the evaluation of the index test (ΔCRT-PLR) and the reference standard (ΔCRT-VE)” |
| Analysis | 14 Methods for estimating or comparing measures of diagnostic accuracy | Item reported: “ ROC curves were built and AUC was expressed with 95% confidence interval calculated with a bootstrap method using 2000 repetitions.” |
|  | 15 How indeterminate index test or reference standard results were handled | Item reported: “cf figure1 “ |
|  | 16 How missing data on the index test and reference standard were handled | Item reported: « CRT was assessed with 4 videos in each of the 3 steps of the study, except for one patient who had only 9 over 12 videos acquisition due to a technical issue, the data were included in the final analysis. Blood gases were missing in 6 patients due to transport or analytical issues.” |
|  | 17 Any analyses of variability in diagnostic accuracy, distinguishing pre-specified from exploratory | **Item not reported** |
|  | 18 Intended sample size and how it was determined | Item reported: ”Sample size calculation was performed using Obuchowsky’s method. Thirty-four patients were needed to detect an area under the ROC curve (ROC_AUC_) of 0.8 with a power of 0.90 and an alpha risk of 0.05. The ratio between responders (R) and non-responders (NR) in our population was hypothesized to be 0.5.” |
| RESULTS |  |  |
| Participants | 19 Flow of participants, using a diagram | Item reported: “cf figure 2 “ |
|  | 20 Baseline demographic and clinical characteristics of participants | Item reported: “ table 1 “ |
|  | 21a Distribution of severity of disease in those with the target condition | Item reported: “cf figure 3 “ |
|  | 21b Distribution of alternative diagnoses in those without the target condition | Item reported: “cf figure 3 “ |
|  | 22 Time interval and any clinical interventions between index test and reference standard | Item reported: « Volume expansion consisted of a 500 ml of lactated ringer administered over 20 minutes. No modification of administration rate or new drug administration occurred during the experimental time.” |
| Test results | 23 Cross tabulation of the index test results (or their distribution) by the results of the reference standard | Item reported: “cf figure 3 |
|  | 24 Estimates of diagnostic accuracy and their precision (such as 95% confidence intervals) | Item reported: ”The area under the ROC curve (AUC) of Δ CRT-PLR was 0.94 [95% CI, 0.87 to 1.0]” |
|  | 25 Any adverse events from performing the index test or the reference standard | Item reported: ”We do not report any adverse events from performing the CRT and the PLR. ” |
| DISCUSSION |  |  |
|  | 26 Study limitations, including sources of potential bias, statistical uncertainty, and generalisability | Item reported: |
|  | 27 Implications for practice, including the intended use and clinical role of the index test | Item reported: |
| OTHER INFORMATION |  |  |
|  | 28 Registration number and name of registry | Item reported:Institutional Review Board (IRB) for human projects (CPP Lyon Sud-Est, ANSM: 2014-A01034-43) and the protocol was published a priori (Clinicaltrial.gov: NCT02248025). |
|  | 29 Where the full study protocol can be accessed | **Item not reported:** |
|  | 30 Sources of funding and other support; role of funders | Item reported: |
